# Advancing PrEP for HIV prevention: innovations and the imperative to preserve comprehensive care

**DOI:** 10.1186/s44263-025-00125-1

**Published:** 2025-01-30

**Authors:** Angel B. Algarin, Cho Hee Shrader

**Affiliations:** 1 Center for Health Promotion and Disease Prevention, Edson College of Nursing and Health Innovation, Arizona State University, Downtown Phoenix Campus, Phoenix, AZ 85004 USA; 2 Edson College of Nursing and Health Innovation, Arizona State University, Downtown Phoenix Campus, Phoenix, AZ USA

The development of pre-exposure prophylaxis has revolutionized HIV prevention, with advancements like long-acting injectables. However, implementation may disrupt essential wraparound services. This comment highlights the need to balance technological progress with comprehensive care models, advocating for policies and strategies that ensure equitable, holistic HIV prevention.

## Background

The development of pre-exposure prophylaxis (PrEP) has revolutionized global HIV prevention, offering a highly effective biomedical strategy to reduce transmission risk. Recent advancements, such as long-acting injectable PrEP, promise to alleviate the burden of daily adherence and frequent clinical interactions, representing a significant step forward in patient-centered care. However, while these innovations address critical barriers such as the aforementioned, they may unintentionally disrupt the provision of ancillary services traditionally integrated into quarterly PrEP visits, such as routine access to healthcare providers, HIV risk reduction counseling, sexually transmitted infection (STI) testing and treatment, behavioral health interventions including risk reduction education, and navigation to wraparound services such as mental health and substance use counseling, and housing, food pantry, and social support programs.

## Advancements in PrEP: a paradigm shift

PrEP has evolved significantly since the 2010 iPrEx trial demonstrated the efficacy of daily oral tenofovir disoproxil fumarate/emtricitabine in reducing HIV acquisition [1]. This pivotal study was followed by the DISCOVER trial in 2019, which introduced a second daily regimen (tenofovir alafenamide/emtricitabine) associated with fewer bone and renal side effects [2]. Most recently, long-acting injectable options have redefined prevention strategies.

In 2020, HIV Prevention Trial Network’s trials 083 and 084 introduced the first injectable PrEP option, cabotegravir, administered every 2 months [3, 4]. The PURPOSE 1 trial in 2024 further expanded options with a twice-yearly injectable lenacapavir for cisgender women, while preliminary PURPOSE 2 results suggest similar efficacy among men (see www.purposestudies.comfor more details). Looking ahead, Gilead has announced plans for a phase 3 trial of an annual PrEP injection [5]. While these innovations address critical adherence challenges, the broader healthcare impacts of reduced clinical interaction must also be carefully considered.

## Unintended consequences: the potential underutilization of ancillary and wraparound services

Historically, PrEP has been delivered alongside comprehensive ancillary healthcare services, including regular HIV and STI screenings, risk-reduction counseling, kidney and lipid monitoring, and consistent provider contact [6], in addition to wraparound services such as case management, mental health and substance use counseling, and housing, food pantry, and social support programs.

There has been a global shift toward national health plans and insurance coverage for PrEP and its associated services, steadily improving affordability and accessibility in many regions. However, transitioning to models with fewer medical or laboratory visits may inadvertently reduce access to these services, potentially widening health inequities. For example, Jenness et al. (2017) demonstrated that biannual STI testing alongside PrEP could prevent approximately 40% of cumulative gonorrhea and chlamydia infections over a decade, while quarterly testing could reduce these infections by an additional 50% [7]. As STIs increase susceptibility to HIV through mucosal disruption, immune changes, and disruption of the genital tract microenvironment [8], reducing STIs may also support reductions in HIV transmission. The implications also extend beyond sexual health outcomes. For example, kidney function assessments, often conducted via a comprehensive metabolic panel (CMP), are not recommended for injectable cabotegravir per the United States Centers for Disease Control and Prevention (CDC) guidelines [6]. While eliminating CMPs may reduce healthcare costs, it risks missing broader health insights, such as early warning signs of liver or metabolic conditions, which are seen more often in communities of color.

## Balancing innovation with comprehensive care

As PrEP delivery evolves, we must balance the benefits of long-acting injectables with the need to preserve ancillary healthcare and wraparound services. Policymakers and researchers should explore scenarios where insurance coverage for ancillary healthcare and wraparound services is maintained, as well as scenarios where it is reduced or eliminated. Proactively planning for these possibilities will offer critical insights for clinical implementation and readiness.

Emerging technologies, such as telehealth, mobile health, and community-based care, may help bridge gaps by delivering STI testing, risk-reduction counseling, and mental health support outside traditional clinical settings. These models should be evaluated alongside new PrEP modalities to ensure holistic care is accessible to all, particularly marginalized populations.

However, this holistic care cannot be effectively delivered through a one-size fits all approach as marginalized communities across the globe continue to face barriers to PrEP uptake and adherence due to medical mistrust and stigma in healthcare settings [9]. Thus, although we urge for the continuation of ancillary and wraparound services, we also urge current programs to critically inspect their delivery of this care to ensure they are respectful, bias-free, and culturally humble.

## Implications for policy and practice

### Policy recommendations

Policymakers must ensure that insurance coverage for ancillary services remains intact, even as PrEP modalities shift, and consider including the provision of essential wraparound services. Policies should expand upon existing services to include mental health and substance use counseling and support housing, food pantry, and social support services, addressing the broader health needs of individuals at risk for HIV.

### Provider training and support

Healthcare providers require training to integrate ancillary services into reduced-visit models. This may include leveraging telehealth, mobile health, and community-based care, for routine screenings and counseling. Training must also consider how to treat each patient with respect while considering their unique intersectional identities.

### Research priorities

Future research should evaluate patient outcomes under reduced-visit models, particularly among marginalized groups. Emerging artificial intelligence technology or intricate electronic health record algorithms may support the identification of patients who may experience poorer outcomes. Investigating how to optimize ancillary healthcare and wraparound services within emerging PrEP frameworks will be critical to inform equitable implementation.

## Conclusions

Long-acting injectable PrEP represents a milestone in HIV prevention, reducing barriers to adherence and expanding accessibility. However, these advancements must not come at the expense of the comprehensive care foundational to PrEP delivery. By proactively addressing these challenges, we can align PrEP innovations with broader public health goals, fostering progress without compromising equity.

## Data Availability

No datasets were generated or analysed during the current study.
